# Supplementary material for: Lentivirus Enables the Detection of Strand-Specific ssDNA Gaps by the DNA Fiber Spreading Assay
Source: Res Sq. 2025 Dec 12:rs.3.rs-8265086. Preprint. [Version 1] doi: 10.21203/rs.3.rs-8265086/v1 (PMC12776476; doi:10.21203/rs.3.rs-8265086/v1)
Supplement: 1 [file NIHPPRS8265086V1-supplement-1.pdf]

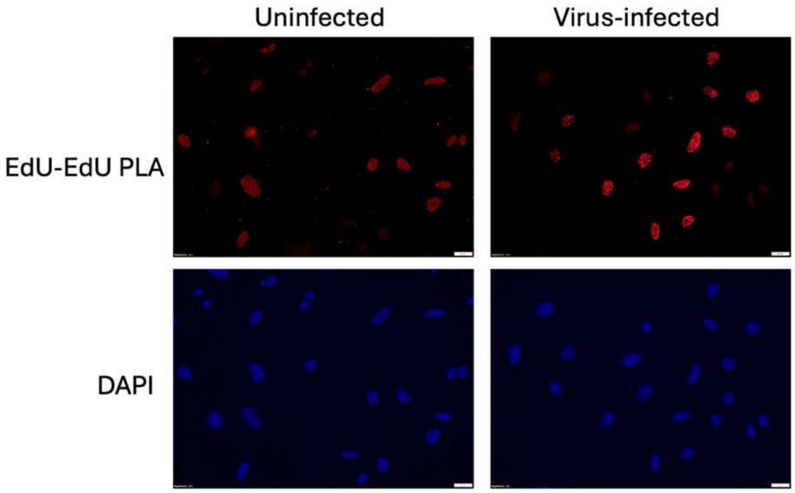

Supplemental Figure 1. Lentiviral infection does not decrease EdU incorporation, related to Figure 6. U2OS cells infected or not with shLUC lentivirus were pulsed labeled four days later with 10 μM EdU for 20 min, and subjected to EdU-EdU PLA assay to assess EdU incorporation level.
